# Biochemical and *in silico* inhibition of bovine and human carbonic anhydrase-II by 1H-1,2,3-triazole analogs

**DOI:** 10.3389/fchem.2022.1072337

**Published:** 2022-11-25

**Authors:** Majid Khan, Satya Kumar Avula, Sobia Ahsan Halim, Muhammad Waqas, Mufarreh Asmari, Ajmal Khan, Ahmed Al-Harrasi

**Affiliations:** ^1^ Natural and Medical Sciences Research Center, University of Nizwa, Birkat Al Mauz, Nizwa, Oman; ^2^ H.E.J Research Institute of Chemistry, International Center for Chemical and Biological Sciences, University of Karachi, Karachi, Pakistan; ^3^ Department of Pharmaceutical Chemistry, College of Pharmacy, King Khalid University, Abha, Saudi Arabia

**Keywords:** synthesis, Suzuki–Miyaura cross-coupling, “click” chemistry, 1H-1,2,3-triazole analogs, carbonic anhydrase-II inhibitory activity, molecular docking studies

## Abstract

A series of 1H-1,2,3-triazole analogs (7a–7d and 9a–9s) were synthesized via “click” chemistry and evaluated for *in vitro* carbonic anhydrase-II (bovine and human) inhibitory activity. The synthesis of intermediates, 7a and 7c, was achieved by using (S)-(-)ethyl lactate as a starting material. These compounds (7a and 7c) underwent Suzuki–Miyaura cross-coupling reaction with different arylboronic acids in 1,4-dioxane, reflux at 90–120°C for 8 h using Pd(PPh_3_)_4_ as a catalyst (5 mol%), and K_2_CO_3_ (3.0 equiv)/K_2_PO_4_ (3.0 equiv) as a base to produce target 1H-1,2,3-triazole molecules (9a–9s) for a good yield of 67–86%. All the synthesized compounds were characterized through NMR spectroscopic techniques. Furthermore, all those compounds have shown significant inhibitory potential for both sources of carbonic anhydrase-II (CA-II). In the case of bCA-II, compounds 9i, 7d, 9h, 9o, 9g, and 9e showed potent activity with IC50 values in the range of 11.1–17.8 µM. Whereas for hCA-II, compounds 9i, 9c, 9o, and 9j showed great potential with IC50 values in the range of 10.9–18.5 µM. The preliminary structure–activity relationship indicates that the presence of the 1H-1,2,3-triazole moiety in those synthesized 1H-1,2,3-triazole analogs (7a–7d and 9a–9s) significantly contributes to the overall activity. However, several substitutions on this scaffold affect the activity to several folds. The selectivity index showed that compounds 9c, 9k, and 9p are selective inhibitors of hCA-II. Kinetics studies showed that these compounds inhibited both enzymes (bCA-II and hCA-II) in a competitive manner. Molecular docking indicates that all the active compounds fit well in the active site of CA-II. This study has explored the role of 1H-1,2,3-triazole-containing compounds in the inhibition of CA-II to combat CA-II-related disorders.

## 1 Introduction

Carbonic anhydrases (EC 4.2.1.1) are zinc metalloenzymes, which serve as therapeutic targets for numerous health issues such as glaucoma, cerebral edema, epilepsy, and various types of cancers (Nocentini and Supuran, 2019). These enzymes mainly catalyze a key physiological reaction, i.e., interconversion of carbon dioxide and bicarbonate to acutely lower the level of carbon dioxide in the blood and other cells of the body (Smith and Ferry, 2000; Khan et al., 2022a). Several other important physiological processes are also regulated by these enzymes, such as trafficking of carbon dioxide between the lungs and other body tissues; maintaining the body homeostasis; biosynthesis reactions such as gluconeogenesis, lipogenesis, bone resorption, calcification, and tumor formation; and other vital pathological or physiological reactions (Ghiasi et al., 2012; Čapkauskaitė et al., 2012; Aslan et al., 2019; Khan et al., 2020; Khan et al., 2021; Aspatwar et al., 2022; Rasool et al., 2022). So far, 16 isozymes of CA with specific functions have been discovered in mammals, such as cytosolic carbonic anhydrases (e.g., CA I-III and CAVII), membrane-bound isozymes (e.g., CA-IV, -IX, -XII, -XIV, and -XV), mitochondrial isozyme (e.g., CA-V), and secreted CA (e.g., CA-VI). Recently, two CA isozymes (viz., CA-IX and -XII) gained attention due to their association with tumors. CA-IX is one of the key enzymes which are controlled by the hypoxia-induced transcription factor (HIF-1α). This membrane-bound enzyme catalyzes the reversible hydration of carbon dioxide to bicarbonate in the extracellular space and plays an important role in the progression of tumor. CA-XII is also implicated in extracellular acidification in many types of tumor (Nocentini and Supuran. 2018). Consequently, CA-IX and -XII are driving factors for tumor growth, invasiveness, proliferation, metastasis, and resistance to radio- and chemotherapy (Supuran et al., 2018; Daunys and Petrikaitė, 2020; Rafiq et al., 2021a; Khan et al., 2022b; Rehman et al., 2022).

Inhibition of CA isoenzyme is important in the treatment of several diseases such as edema, glaucoma, obesity, cancer, epilepsy, and osteoporosis. It makes these enzymes a valuable therapeutic target in the treatment of primary tumor growth, metastasis, and the reduction of cancer stem cell population (Beyza Öztürk Sarıkaya et al., 2010; Nocentini et al., 2019; Hashmi et al., 2021; Rehman et al., 2022).

Compounds with the 1H-1,2,3-triazole moiety are crucially important in pharmaceuticals, agrochemicals, and medicinal chemistry (Abdel-Wahab et al., 2012; Avula et al., 2021a). The 1H-1,2,3-triazole group is significant in organic chemistry due to their wide range of applications in bio-medicinal, biochemical, material sciences, and pharmaceuticals (Singh et al., 2010). 1H-1,2,3-triazole-bearing molecules have undergone substantial growth over the past few decades (Thirumurugan et al., 2013). Those compounds have wide industrial applications such as dyes, photostabilizers, photographic materials, corrosion inhibitors, and agrochemicals (Green et al., 1995). Therefore, the synthesis of 1H-1,2,3-triazole derivatives could be a topic of special interest for synthetic chemists because of their biological, medicinal, and industrial applications. Due to this reason, great effort has been directed toward developing new synthetic methodologies for 1H-1,2,3-triazole moieties.

**FIGURE 1:**
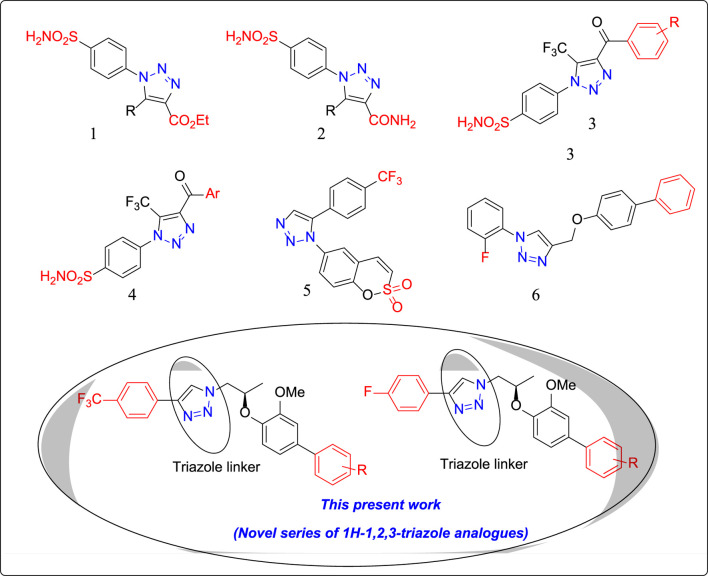
Structures of few clinically used 1H-1,2,3-triazole analogues as potential CA-II inhibitors. **1:** 1H-1,2,3-triazol-1-yl) benzenesulfonamide **2:** 1H-1,2,3-triazole-4-carboxamide **3:** 1*H*-1,2,3-triazol-1-yl) benzenesulfonamide **4:** 1H-1,2,3-triazol-1-yl) benzenesulfonamide **5:** 1H-1,2,3-triazol-1-yl) benzo [e][1,2] oxathiine 2,2-dioxide **6**: 4-{[(1,1′-biphenyl)-4-yloxy] methyl}-1-(2-fluorophenyl)-1H-1,2,3-triazole.

Recent literature studies (Kumar et al., 2018; Sharma et al., 2019) have reported the superior carbonic anhydrase inhibitory potential of 1H-1,2,3-triazole ring-containing heterocycles (Figure 1). In continuation of our research work on 1H-1,2,3-triazole analogues (Avula et al., 2018; Avula et al., 2019; Avula et al., 2021a; Avula et al., 2021b), we, herein, report the synthesis of a new series of novel 1H-1,2,3-triazole analogues (**7a**–**7d** and **9a**–**9s**) via “click” chemistry and their *in vitro* CA-II inhibitory activities for the first time.

“Click” chemistry refers to a group of reactions that are fast, simple to use, easy to purify, versatile, region specific, and give a high product yield. Among several reactions that fulfill the criteria, Huisgen 1,3-dipolar cycloaddition of azides and terminal alkynes has emerged as a frontrunner. It has applications in a wide variety of research areas, including material sciences, polymer chemistry, and pharmaceutical sciences (Thi et al., 2016; Nabih et al., 2020; Kaur et al., 2021).

The current study aimed to thoroughly investigate the biological activities of novel synthesized compounds (**7a**–**7d** and **9a**–**9s**) as effective CA inhibitors. Their inhibitory effect on bovine (bCA-II) and human (hCA-II) enzymes was examined, and molecular modeling was performed to predict their possible mode of binding inside the active sites of bCA and hCA.

## 2 Materials and methods

### 2.1 Chemistry

Reagents were obtained from Sigma-Aldrich, Germany. Silica gel was used for column chromatography with 100–200 mesh. All solvents were purified by following the standard procedure. Thin-layer chromatography (TLC) was performed on silica gel F_254_ pre-coated plates. UV-light and I_2_ stain were used to visualize the spots. The ^1^H and ^13^C NMR spectra were recorded on an NMR spectrometer (Bruker: 600 MHz for ^1^H, 150 MHz for ^13^C, and 564 MHz for ^19^F) using CDCl_3_ as a solvent. High-resolution electrospray ionization mass spectra (HR-ESI-MS) were recorded on the Agilent 6530 LC Q-TOF instrument. Organic extracts and solutions of pure compounds were dried over anhydrous MgSO_4_.

### 2.2 Biological enzyme inhibition

#### 2.2.1 Carbonic anhydrase assay

CA inhibitory activity was determined according to the spectrophotometric method described in the literature (Rafiq et al., 2021b). Total assay volume comprises 140 µl of HEPES-Tris buffer, 20 µl of bCA-II solution (0.1 mg/ml HEPES-Tris buffer), 20 µl of the test compound (prepared in DMSO), and 20 µl *p*-NPA (0.7 mM, methanol). First, the test compound and enzyme were incubated for 15 min at 25°C; after incubation, in quick sequence, substrate was added to each experiment in 96-well plates, which were placed in the ^x^MARK microplate spectrophotometer (Bio-Rad, United States), and the absorbance was set to 400 nm with 1 min interval time. The activity of the controlled compound was taken as 100%. All experiments were carried out thrice for each used concentration, and the results were presented as a mean of the triplicate, and % inhibition of each compound was calculated using the following formula:

$$\%\;Inhibition=100-\frac{Absorbance\;of\;test\;compound}{Absorbance\;of\;control}\;x\;100.$$

#### 2.2.2 Kinetics protocol

For mechanistic studies, both human and bovine isozymes (0.1 mg/ml/well) were incubated with different concentrations of test compounds (with respect to their determined IC_50_ values; two concentrations higher and two concentrations lower than their respective IC_50_) for 15 min at 25°C. Soon after the incubation, various concentrations of substrate were added to the reaction medium (01–0.8 mM). The enzyme activity was measured under steady-state conditions by observing changes in absorbance for 30 min at 400 nm on the microtiter plate reader (Bio-Rad X-Mark^Tm^ molecular spectrometer, United States). The results were analyzed and processed by Grafit 7 software (Erithacus Software Limited, United Kingdom). Different kinetic parameters of enzymes (e.g., *Vmax*, *Km*, *Vmaxapp*, and *Kmapp*) and values of correlation coefficients, intercepts, slopes, and their standard error mean were calculated by the linear regression graph by Grafit 7. Linear regression analysis was used to calculate the “best fit” line or curve through a dataset by minimizing the deviation of the data from the curve.

#### 2.2.3 Docking protocol

*In silico* docking is widely used to explore the binding mechanism of biologically active molecules. The X-ray structures of bCA-II (PDB ID: 1V9E, resolution = 1.95 Å) (Saito et al., 2004) and hCA-II (PDB code: 1BN1, resolution = 2.1 Å) (Boriack-Sjodin et al., 1998) were downloaded from the Protein Data Bank (www.rcsb.org) for docking studies. Chain A was retained if more than one chain was present in the structure. The Molecular Operating Environment (MOE) (MOE version 2020.0901) was used in docking. Initially, protein structures were treated using the Protein Preparation Wizard of MOE by adding hydrogen atoms and making partial charges on residues with the AMBER12:EHT forcefield. Subsequently, the protein structures were subjected to short energy minimization and geometric optimization with the AMBER12:EHT forcefield with an RMSD gradient of 0.1 kcal mol^−1^Å^−1^. The structures of ligands were prepared by ChemDraw and minimized by the MOE with an RMSD gradient of 0.1 kcal∙mol^−1^Å^−1^ and MMFF94x forcefield. For docking, the Triangle Matcher placement method and London dG scoring function were applied. After docking, 30 docked possess of each compound were saved, and the best scoring docked pose was visualized.

## 3 Results and discussion

### 3.1 Synthesis of 1H-1,2,3-triazole analogues (7a–7d)

**SCHEME 1:**
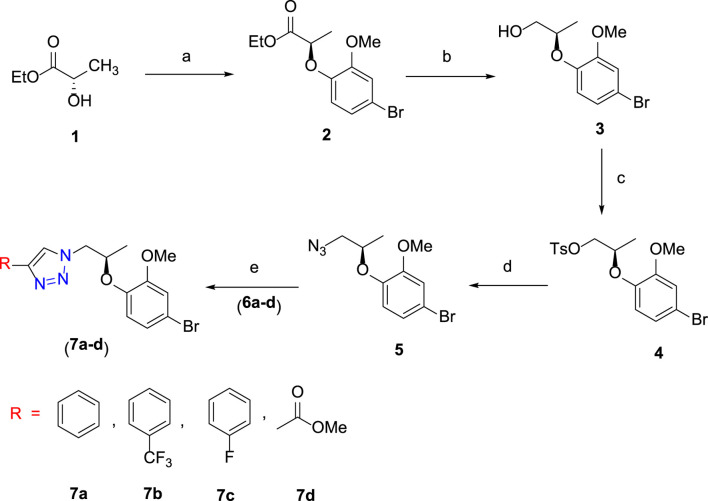
Reagents and conditions: **(A)** 4-bromo-2-methoxyphenol, PPh_3_, DIAD, dry THF, 0 ^°^C to room temperature, 3 h, 86%; **(B)** DIBAL-H, dry DCM, 0°C to room temperature, 3 h, 90%; **(C)** TsCl, Et_3_N, dry DCM, DMAP, 0°C to room temperature, 5 h, 95%; **(D)** NaN_3_, DMF, 70°C, 3 h, 78%; **(E)** alkyne derivative (**6a**–**6d**), CuI, Et_3_N, MeCN, room temperature, 3 h, **7a**–**7d** (74–80%).

The synthetic scheme of a new series of 1H-1,2,3-triazole analogues via “click” chemistry **(7a-7d)** is depicted in Scheme 1. In an initial step, Mitsunobu reaction was carried out between compound **1** and 4-bromo-2-methoxyphenol in the dry THF solvent using diisopropylazodicarboxylate (DIAD), which produced desired compound **2** (86%). In the next step, compound **2** underwent reduction by using DIBAL-H from 0°C to room temperature and in dry DCM, to produce desired compound **3** (90%). In compound **3**, a free –OH group was protected with *p*-toluenesulfonyl chloride in the presence of triethylamine (Et_3_N) and in dry DCM at 0°C to room temperature to give compound **4**, which was treated with NaN_3_ in DMF at 70°C to generate desired compound **5**.

The final step was carried out using “click” chemistry (Avula et al., 2018; Avula et al., 2019) where a 1,3-dipolar cycloaddition reaction occurred between alkyne derivatives **6a**–**6d** and compound **5** in the presence of Hunig’s base and CuI in the acetonitrile solvent to produce target 1H-1,2,3-triazole analogues **(7a**–**7d)**.

### 3.2 Synthesis of cross-coupled 1H-1,2,3-triazole analogs (9a–9s)

**SCHEME 2:**
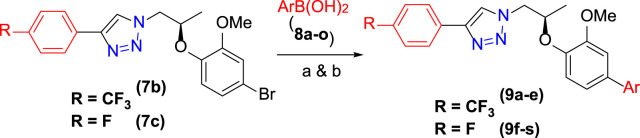
Synthesis of **9a**–**9s**: reagents and conditions: **(A) 7b** (1.0 equiv), **8a**–**8e** (1.2–1.5 equiv), Pd(PPh_3_)_4_ (5 mol%), K_2_PO_4_ (3.0 equiv), 1,4-dioxane, 90–110°C, 8 h. **(B) 7c** (1.0 equiv), **8f**–**8s** (1.5 equiv), Pd(PPh_3_)_4_ (5 mol%), K_2_CO_3_ (3.0 equiv), 1,4-dioxane, 120°C, 8 h.

The synthetic scheme of cross-coupled 1H-1,2,3-triazole analogues (**9a**–**9s**) is depicted in Scheme 2. The final step was the functionalization of the aryl bromide moiety by the Suzuki–Miyaura cross-coupling reaction (Hassan et al., 2015; Hassan et al., 2017) of **7b** and **7c** with different arylboronic acids (**8a**–**8o)** in 1,4-dioxane solvent, refluxed at 90–120°C for 8 h to afford a novel series of 1H-1,2,3-triazole analogues (**9a**–**9s**) for a good yield (67–86%) (Table 1). The best yield was obtained in 1,4-dioxane solvent, refluxed at 90–120°C for 8 h using Pd(PPh_3_)_4_ as a catalyst (5 mol%) and K_2_CO_3_ (3.0 equiv)/K_2_PO_4_ (3.0 equiv) as a base. The structures of newly synthesized compounds (**9a**–**9s**) were confirmed by NMR spectroscopic techniques.

**TABLE 1:** Synthesis of cross-coupled 1H-1,2,3-triazole analogues (9a–9s).

Compound (7b and 7c	Reagent (8)	Product (9a–9s)	Ar	Yield (%)^a^
**7b**	A	A	4-MeC_6_H_4_	73
**7b**	b	B	4-MeOC_6_H_4_	67
**7b**	c	C	2-CF_3_C_6_H_4_	77
**7b**	d	D	4-FC_6_H_4_	80
**7b**	e	E	4-NO_2_C_6_H_4_	82
**7c**	f	F	Ph	76
**7c**	a	G	4-MeC_6_H_4_	73
**7c**	b	H	4-MeOC_6_H_4_	69
**7c**	e	I	4-NO_2_C_6_H_4_	85
**7c**	g	J	4-ClC_6_H_4_	82
**7c**	h	K	4-NCC_6_H_4_	79
**7c**	d	L	4-FC_6_H_4_	84
**7c**	i	M	3,5-(F_3_C)_2_C_6_H_3_	80
**7c**	j	N	4-CHOC_6_H_4_	77
**7c**	k	O	2,6-(F)_2_C_6_H_3_	82
**7c**	l	P	2,3,4-(F)_3_C_6_H_2_	86
**7c**	m	Q	2-C_10_H_7_	79
**7c**	n	R	4-AcC_6_H_4_	82
**7c**	o	S	2-C_8_H_5_S	78

^a^ Yield refers to pure isolated products.

#### 3.2.1 General reaction procedure for cross-coupled 1H-1,2,3-triazole analogs (9a–9s)

1,4-dioxane (5 ml per 1 mmol) solution of **7b** or **7c** (1.0 equiv), K_2_CO_3_ (3.0 equiv)/K_2_PO_4_ (3.0 equiv), Pd (PPh_3_)_4_ (5 mol%), and arylboronic acid **8a**–**8s** (1.2 or 1.5 equiv) were stirred at 90–120°C for 8 h. After cooling down to 20°C, H_2_O was added. The organic and the aqueous layers were separated, and the latter was extracted with CH_2_Cl_2_ (15 × 3 ml). The combined organic layer was dried over anhydrous MgSO_4_ and filtered, and the filtrate was concentrated *in vacuo*. The residue was purified by column chromatography (EtOAc/Hexane 9:1) to give pure cross-coupled products **9a**–**9s**.

### 3.3 *In vitro* inhibition of bCA-II by 1H-1,2,3-triazole analogues (7a–7d and 9a–9s)

The present study focused on the *in vitro* inhibition of bovine and human CA-II (bCA-II and hCA-II) by triazole derivatives (**7a**–**7d**, and **9a**–**9s**) and their binding interactions with the enzyme active site. As the efficient and safer CA inhibitors have been urgently required to expand therapeutic regimes for glaucoma and epilepsy. Compounds **7a**–**7d** and **9a**–**9s** showed IC_50_ values in the range of 11.1–88.4 µM. Compound **9i** (IC_50_ = 11.1 ± 0.2 µM) is the most potent inhibitor of bCA-II in the current series. Similarly, **7d** (IC_50_ = 12.3 ± 0.4 µM), **9h** (IC_50_ = 12.9 ± 1.0 µM), **9o** (IC_50_ = 14.1 ± 0.2 µM), **9g** (IC_50_ = 16.4 ± 0.4 µM), **9e** (IC_50_ = 17.8 ± 0.8 µM), **7c** (IC_50_ = 20.4 ± 0.3 µM), **7b** (IC_50_ = 20.6 ± 0.3 µM), and **7a** (IC_50_ = 21.4 ± 0.1 µM) showed potent activity as compared to the standard drug, acetazolamide (IC_50_ = 18.6 ± 0.4 µM). Whereas **9a** (IC_50_ = 25.7 ± 0.2 µM), **9d** (IC_50_ = 28.8 ± 0.4 µM), and **9r** (IC_50_ = 29.0 ± 1.0 µM) are moderately active against bCA-II, and **9b** (IC_50_ = 39.1 ± 0.3 µM), **9m** (IC_50_ = 41.0 ± 0.4 µM), and **9f** (IC_50_ = 88.4 ± 0.3 µM) are the least active against bCA-II (Table 2).

**TABLE 2:** *In vitro* inhibition of bCA-II and hCA-II by 7a–7d and 9a–9s.

Compound	bCA-II	hCA-II
	IC50 ± S.E.M. (µM)	IC50 ± S.E.M. (µM)
7a	21.4 ± 0.1	35.3 ± 0.6
7b	20.6 ± 0.3	45.1 ± 0.5
7c	20.4 ± 0.3	22.8 ± 0.2
7d	12.3 ± 0.4	25.1 ± 0.4
9a	25.7 ± 0.2	21.0 ± 1.0
9b	39.1 ± 0.3	22.4 ± 0.4
9c	Inactive	12.5 ± 0.6
9d	28.8 ± 0.4	Inactive
9e	17.8 ± 0.8	Inactive
9f	88.4 ± 0.3	Inactive
9g	16.4 ± 0.4	Inactive
9h	12.9 ± 1.0	Inactive
9i	11.1 ± 0.2	10.9 ± 1.0
9j	Inactive	18.5 ± 0.4
9k	Inactive	21.3 ± 0.8
9l	Inactive	Inactive
9m	41.0 ± 0.4	27.4 ± 0.9
9n	22.4 ± 0.4	23.0 ± 0.3
9o	14.1 ± 0.2	13.6 ± 1.0
9p	Inactive	19.3 ± 0.2
9q	Inactive	Inactive
9r	29.0 ± 1.0	Inactive
9s	Inactive	Inactive
Acetazolamide	18.6 ± 0.4	19.5 ± 1.0

#### 3.3.1 Kinetics studies of most potent compounds against bCA-II and hCA-II

**FIGURE 2:**
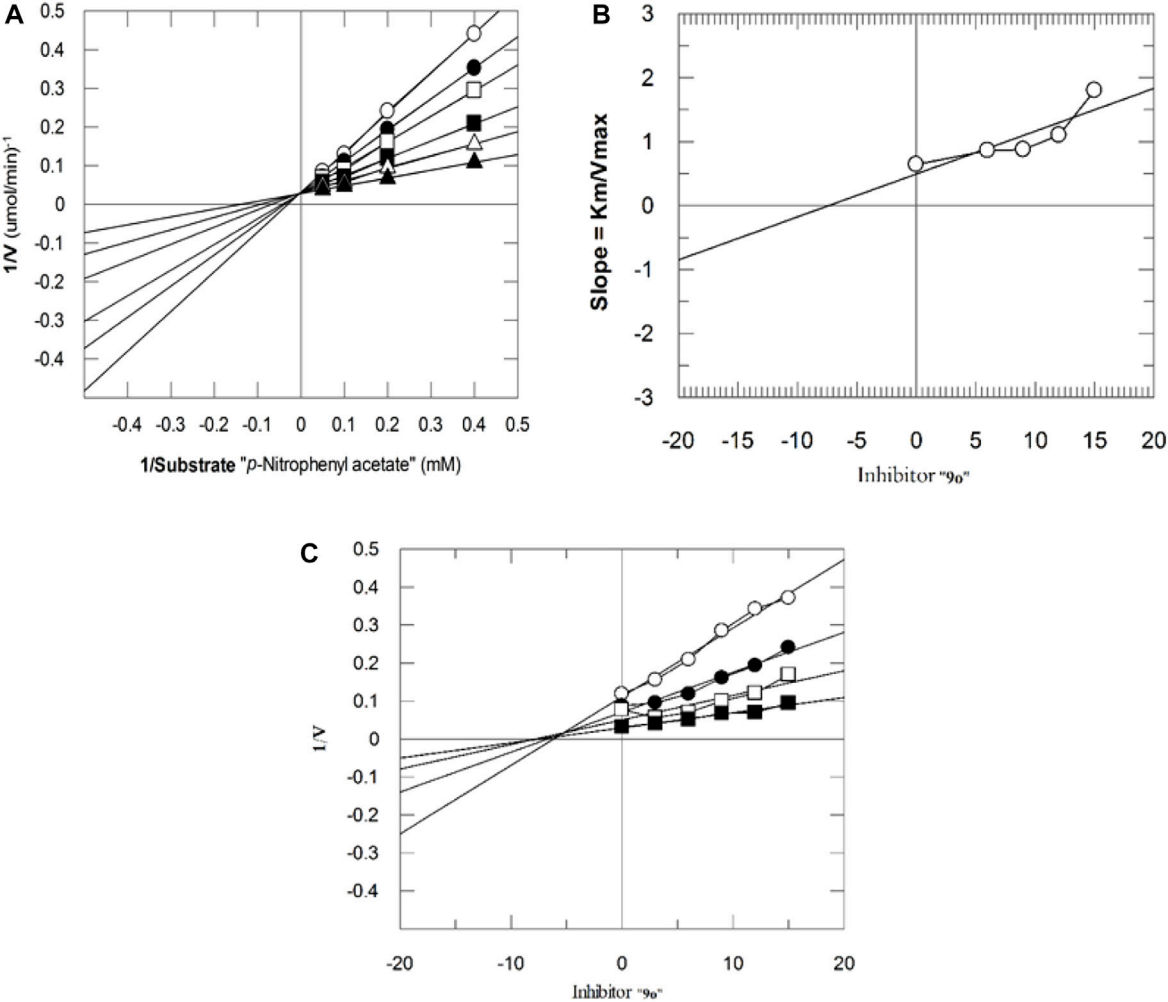
Inhibition of hCA-II by **9o**. **(A)** Lineweaver–Burk plot of the reciprocal of the rate of reaction (velocities) vs*.* reciprocal of the substrate (p-nitrophenyl acetate) in the absence (▲), 6 *µ*M (∆), 9 *µ*M (■), 12 *µ*M (**□**), 15 *µ*M (●), and 18 *µ*M (○) of **9o**. **(B)** Secondary replot of the Lineweaver–Burk plot between the slopes of each line on the Lineweaver–Burk plot vs*.* different concentrations of **9o**. **(C)** Dixon plot of the reciprocal of the rate of reaction (*velocities*) vs*.* different concentrations of **9o**.

**FIGURE 3:**
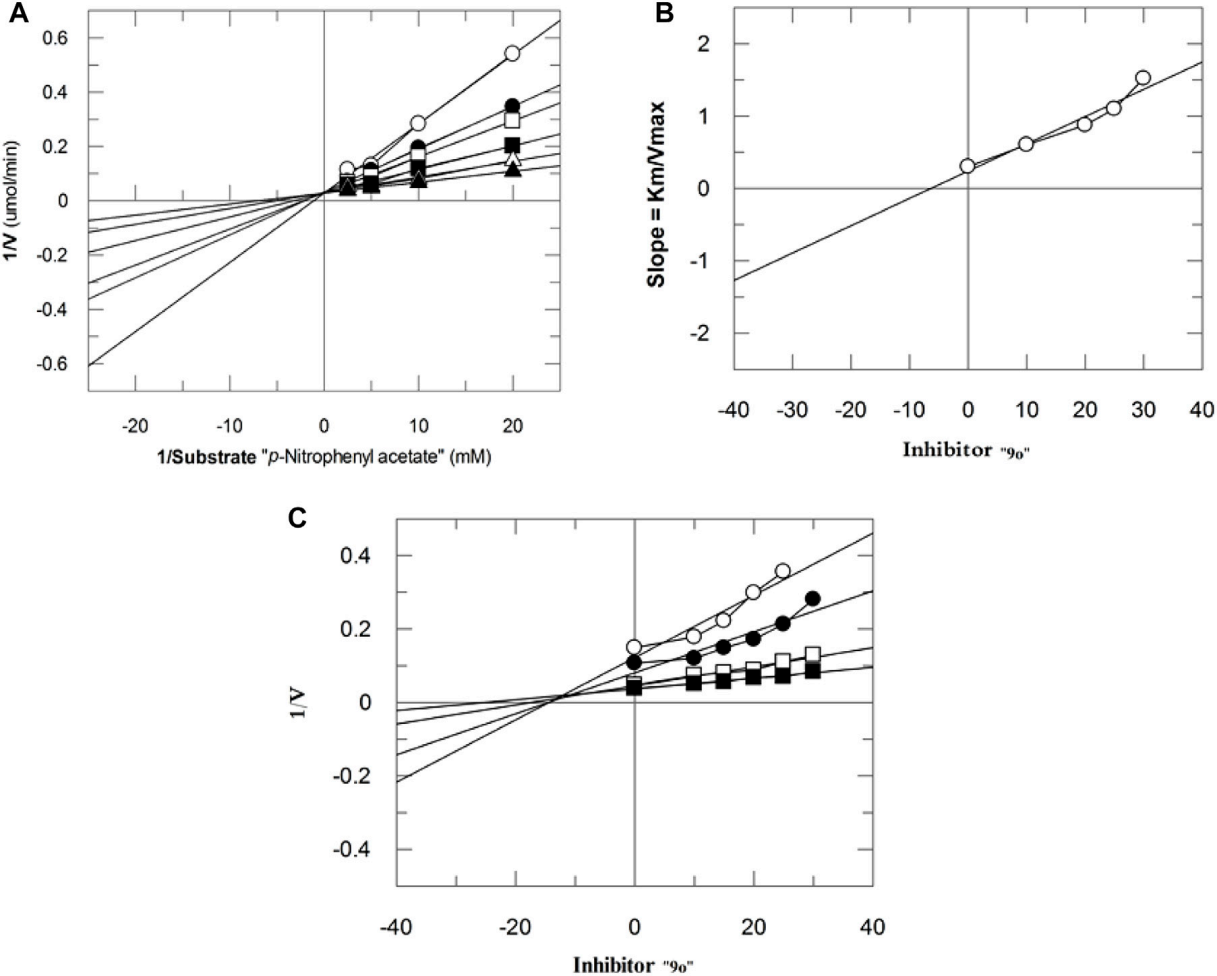
Inhibition of bCA-II by **9o**. **(A)** Lineweaver–Burk plot of the reciprocal of the rate of reaction (velocities) vs*.* reciprocal of the substrate (p-nitrophenyl acetate) in the absence (▲), 7 *µ*M (∆), 10 *µ*M (■), 13 *µ*M (**□**), 16 *µ*M (●), and 19 *µ*M (○) of **9o**. **(B)** Secondary replot of Lineweaver–Burk plot between the slopes of each line on the Lineweaver–Burk plot vs*.* different concentrations of **9o**. **(C)** Dixon plot of the reciprocal of the rate of reaction (*velocities*) vs*.* different concentrations of **9o**.

The mechanism of action of potent compounds was studied in a concentration-dependent manner against both the targeted enzymes. Therefore, compounds 9i and 9o were selected for kinetics studies (Table 3). These compounds (9i and 9o) showed competitive inhibition of both enzymes. 9i and 9o inhibited bCA-II with a dissociation constant (Ki) of 9.4 ± 0.3 and 11.3 ± 0.17 µM, respectively. Similarly, for hCA-II, the dissociation constant (Ki) of 9i and 9o is 8.1 ± 0.04, and 6.5 ± 0.19 µM, respectively. The Lineweaver–Burk plot of 9o for hCA-II and bCA-II is, respectively, presented in Figure 2 and Figure 3.

**TABLE 3:** Kinetic results of compounds **9i** and **9o** for enzymes bCA-II and hCA-II.

Compound	Ki ± SEM (µM)	Km (mM)	Km_app_ (mM)	Vmax (µmol/min)^−1^	Vmax_app_ (µmol/min)^−1^	Type of inhibition
bCA-II						
**9i**	9.4 ± 0.3	3.1	5.6	23.2	23.2	Competitive
**9o**	11.3 ± 0.17	3.2	4.9	24.6	24.6	Competitive
hCA-II						
**9i**	8.1 ± 0.04	2.6	3.9	26.1	26.1	Competitive
**9o**	6.5 ± 0.19	2.4	4.2	25.3	25.3	Competitive

**Vmax** = Maximum velocity of the enzyme in the absence of inhibitor.

**Vmax**_**ap**p_ = Maximum velocity of the enzyme in the presence of inhibitor.

**Km** = Michaelis–Menten constant in the absence of inhibitor.

**Km**_**app**_ = Michaelis–Menten constant in the presence of inhibitor.

#### 3.3.2 Analysis of binding interactions and the structure–activity relationship (SAR) against bCA-II

**FIGURE 4:**
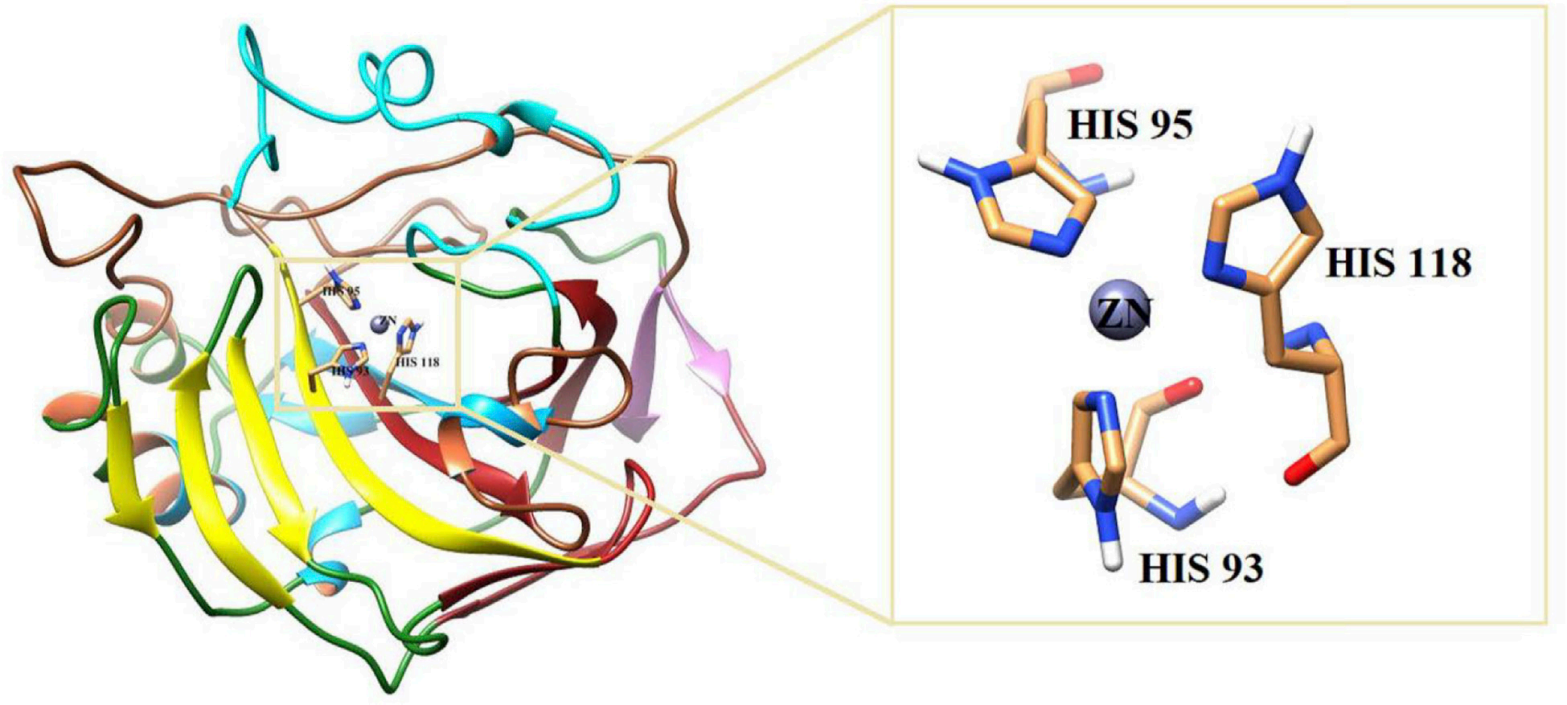
3D-structure of bCA is shown. The active site is highlighted in the box.

All the active compounds were docked at the active site of bCA**-**II (Figure 4), where compounds showed significant binding interactions. An interesting correlation was obtained from the detailed analysis. The compounds bearing nitro oxygen, benzaldehyde, and thiophenone groups significantly interacted with the metallo center, “Zn” ion, in the active site. Whereas other derivatives showed contact with active site residues through their triazole moiety. The most active compound **9i** was chelated with a Zn ion in the active site via its nitro oxygen; in addition, **9i** mediated hydrogen bonds (H-bond) with two water molecules (WAT462 and WAT435) and side chain of Gln91. The bond distances are given in Table 4. Similarly, **7d** interacted with the Zn atom, a water molecule (WAT488), and with the side chain of Gln91. However, compounds **9h**, **9o**, and **9g** lost interactions with Zn, while retained their interactions with the active site residues. Compound **9h** mediated H-bonds with the side chain of Asn66, Gln91, and Thr199, and two water molecules (WAT279 and WAT435). Whereas **9o** formed a halogen bond with the side chain of His93 through its fluorine atom and mediated H-bonds with the side chains of Thr198 and Gln91, and WAT435. Similarly, **9g** also formed H-bonds with the side chain Gln91, WAT279 and WAT493; furthermore, **9g** was stabilized by the π–π stacking interaction by the phenyl ring of Phe129. Compound **9e** was chelated with the Zn ion through its nitro oxygen and formed H-bonds with the side chain of Gln91 and WAT279. The binding mode of **7c** shows that the compound mediated H-bonds with the side chains of Thr198 and Gln91, and WAT279. In contrast, **7b** interacted with the Zn atom through its acetophenone-substituted carbonyl moiety and made H-bonds with the side chain of Thr198 and WAT466. The docked orientation of **7a** revealed that **7a** interacted with the side chains of Gln91 and Asn66 and WAT466 through H-bonds. Compound **9r** showed contact with the side chain of Gln91 and WAT310 (2.32 and 2.27 Å), respectively.

**TABLE 4:** Binding analysis of active 1H-1,2,3-triazole analogues in the active site of bCA-II.

Compound	Binding interactions				
	Score (Kcal/mol)	Ligand atoms	Receptor atom	Bond type	Distance (Å)
9i	−5.45	O30	ZN	Ionic	1.93
		O31	ZN	Ionic	2.34
		O12	WAT462	HBA	3.07
		O5	WAT435	HBA	2.53
		O5	NE2-GLN91	HBA	2.30
7d	−5.76	O20	ZN	Ionic	1.44
		O5	NE2-GLN91	HBA	2.14
		O5	WAT488	HBA	0.88
9h	−5.38	O12	ND2-ASN66	HBA	2.54
		O5	NE2-GLN91	HBA	2.85
		N16	OG1-THR198	HBA	2.15
		N17	WAT279	HBA	2.19
		N5	WAT435	HBA	2.94
9o	−5.24	F1	NE2-HIS93	Halogen	1.82
		N17	OG1-THR198	HBA	2.10
		N5	NE2-GLN91	HBA	2.84
		O5	WAT435	HBA	2.78
9g	−5.04	6-ring	6-ring-PHE129	π–π	2.78
		O12	NE2-GLN91	HBA	2.90
		N16	WAT279	HBA	1.66
		N17	WAT493	HBA	2.13
9e	−5.56	O30	ZN	Ionic	2.26
		O31	WAT279	HBA	2.09
		O12	NE2-GLN91	HBA	2.93
7c	−4.54	N17	OG1-THR198	HBA	2.83
		N1	NE2-GLN91	HBA	2.10
		N16	WAT279	HBA	2.79
7b	−5.03	O12	ZN	Ionic	2.37
		N1	OG1-THR198	HBA	2.10
		N17	WAT466	HBA	2.68
7a	−4.58	O12	NE2-GLN91	HBA	2.38
		O12	ND2-ASN66	HBA	1.63
		N17	WAT466	HBA	2.46
9n	−4.84	O30	ZN	Ionic	1.68
		O12	NE2-GLN91	HBA	2.88
9a	−4.68	O5	NE2-GLN91	HBA	2.86
		O20	WAT279	HBA	2.34
		N16	ND2-ASN66	HBA	2.29
9d	−4.54	N1	NE2-GLN91	HBA	2.71
		N16	WAT435	HBA	1.79
9r	−4.63	N1	NE2-GLN91	HBA	2.32
		N16	WAT310	HBA	2.27
9b	−3.95	6-ring	6-ring-PHE129	π–π	2.74
		N17	WAT435	HBA	1.70
9f	−3.84	N16	NE2-HIS93	HBA	2.16
9m	−4.76	O12	ND2-ASN66	HBA	2.60
		N17	WAT310	HBA	1.98

HBA = hydrogen bond acceptor

**FIGURE 5:**
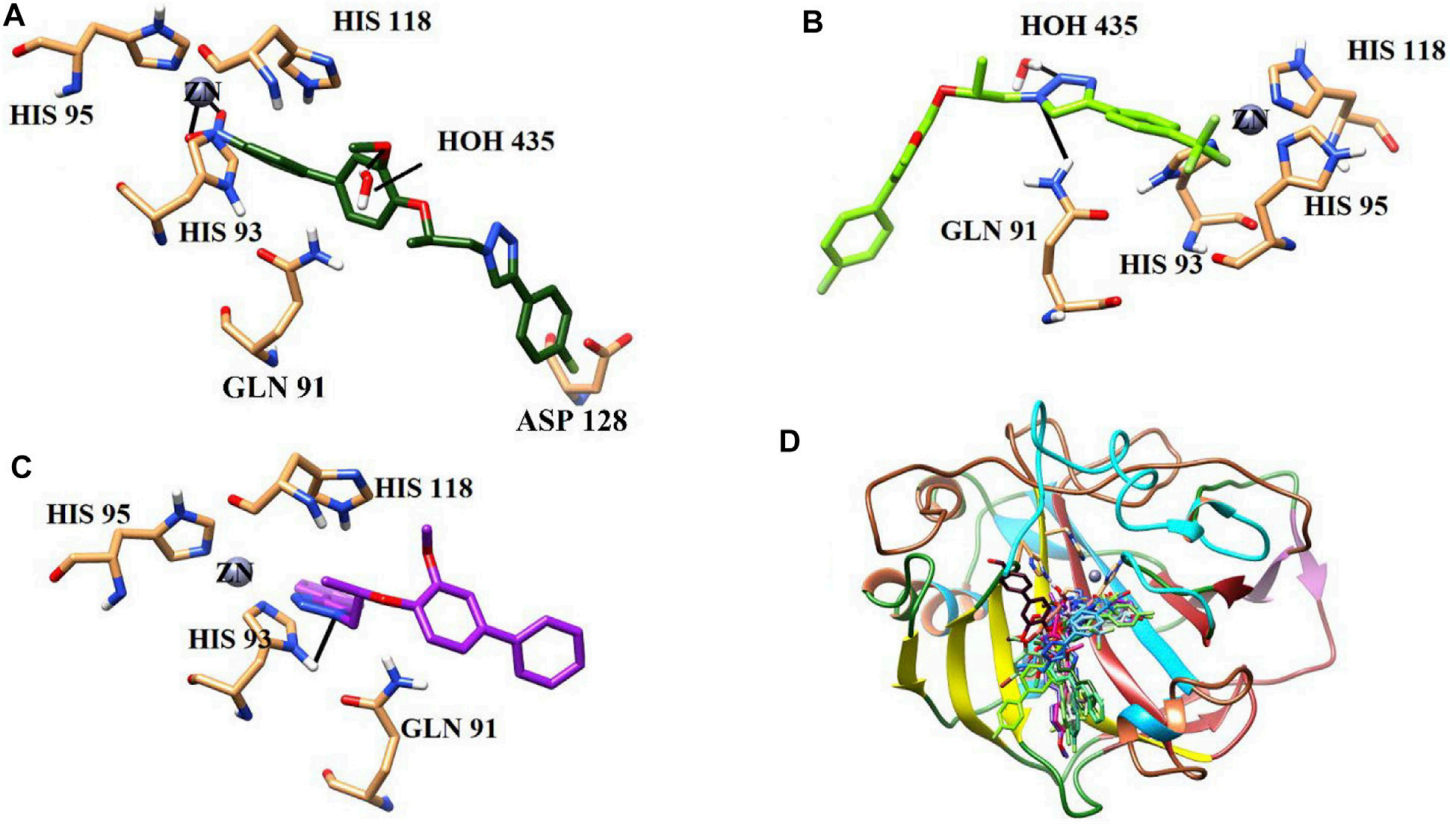
Binding orientations of **(A)** most active compound **9i** (dark green stick model), **(B)** moderate active **9d** (light green stick model), **(C)** least active **9f** (purple stick model), and **(D)** all the active compounds are shown in the active site of bCA. The active site residues are shown in coral sticks, and H-bonds are depicted in black lines.

The binding modes of moderate active compounds **9a**, **9d**, and **9r** demonstrated that all those molecules mediated H-bonds with the side chain of Gln91; however, **9a** also interacted with the side chain of Asn66 and WAT279, whereas **9d** and **9r** formed H-bonds with WAT435 and WAT310. On the other hand, the least active compounds, **9b**, **9f,** and **9m** formed less H-bonds and hydrophobic interactions within the active site. The phenyl ring of Phe129 provided π–π stacking interactions to **9b** which also formed an H-bond with WAT435. Similarly, **9f** formed an H-bond with the side chain of His93, and **9m** made H-bonds with the side chain of Asn66 and WAT310. The detailed binding interactions of the active compounds are tabulated in Table 4, and the binding modes are depicted in Figure 5.

### 3.4 *In vitro* inhibition of hCA by 7a–7d and 9a–9s

In the *in vitro* testing, compound **9i** (IC_50_ = 10.9 ± 1.0 µM) was retrieved as the most potent inhibitor of hCA-II, followed by **9c** (IC_50_ = 12.5 ± 0.6 µM), **9o** (IC_50_ = 13.6 ± 1.0 µM), **9j** (IC_50_ = 18.5 ± 0.4 µM), **9p** (IC_50_ = 19.3 ± 0.2 µM), **9a** (IC_50_ = 21.0 ± 1.0 µM), **9k** (IC_50_ = 21.3 ± 0.8 µM), **9b** (IC_50_ = 22.4 ± 0.4 µM), **7d** (IC_50_ = 25.1 ± 0.4 µM), and **9n** (IC_50_ = 23.0 ± 0.3 µM). Whereas compounds **7c** (IC_50_ = 22.8 ± 0.2 µM) and **9m** (IC_50_ = 27.4 ± 0.9 µM) demonstrated moderate inhibition, while **7a** (IC_50_ = 35.3 ± 0.6 µM) and **7b** (IC_50_ = 45.1 ± 0.5 µM) showed weak inhibitory activity against hCA as compared to acetazolamide (IC_50_ = 19.5 ± 1.0 µM). The results are given in Table 2.

#### 3.4.1 Analysis of binding interactions and the structure–activity relationship (SAR) against hCA-II

The current library of compounds was also evaluated against hCA-II to understand the specificity of these compounds. Among all the compounds, **9i** (IC_50_ = 10.9 ± 1.10 µM) showed the highest inhibitory potential for hCA-II. The binding analysis of **9i** revealed that it mediates significant interaction with a Zn ion and active site residues. The nitro group of **9i** formed an ionic interaction with the Zn atom and bidentate interactions with the side chain of Asn62. Whereas compounds **9c** and **9o** interacted with the side chains of Asn62, Gln92, Thr199, His96, and Thr200. **9c** mediated H-bonds with the side chains of Asn62 and Gln92 and further formed a halogen bond with the side chain of Thr199. Similarly, **9o** formed H-bonds with the side chains of His96 and Thr200 through its methoxybenzene and triazole moieties, respectively. The triazole nitrogen of **9j** formed an ionic bond with Zn and was further stabilized by the side chain of Gln92 through H-bonds; in addition, this compound formed a halogen bond with the side chain of Thr199. Compounds **9p**, **9a**, **9k**, and **9b** were stabilized by the side chains of Thr200, Asn62, Gln92, Thr199, and His96. The methoxyethane moiety of **9p** mediated a H-bond with a side chain of Thr200 and a π–cation interaction with the side chain of Thr199. Whereas **9a** mediated multiple H-bonds with the side chains of Asn62 and Gln92 and a halogen bond with the side chain of Thr199 through its fluoro group. Similarly, **9k** displayed a halogen bond with the side chain of His96 and stabilized by the side chains of Thr199 and Thr200 through H-bonds. Similarly**,** compound **9b** was stabilized by the side chains of Asn62 and His64 by H-bonds and mediated a π–cation interaction with the side chain of Thr199. Compounds **7d** and **9n** formed ionic interactions with Zn ions and H-bonds with the side chains of Thr199, Gln92, and Thr200. Additionally, **9n** also demonstrated a π–cation bond with the side chain of Gln92.

**FIGURE 6:**
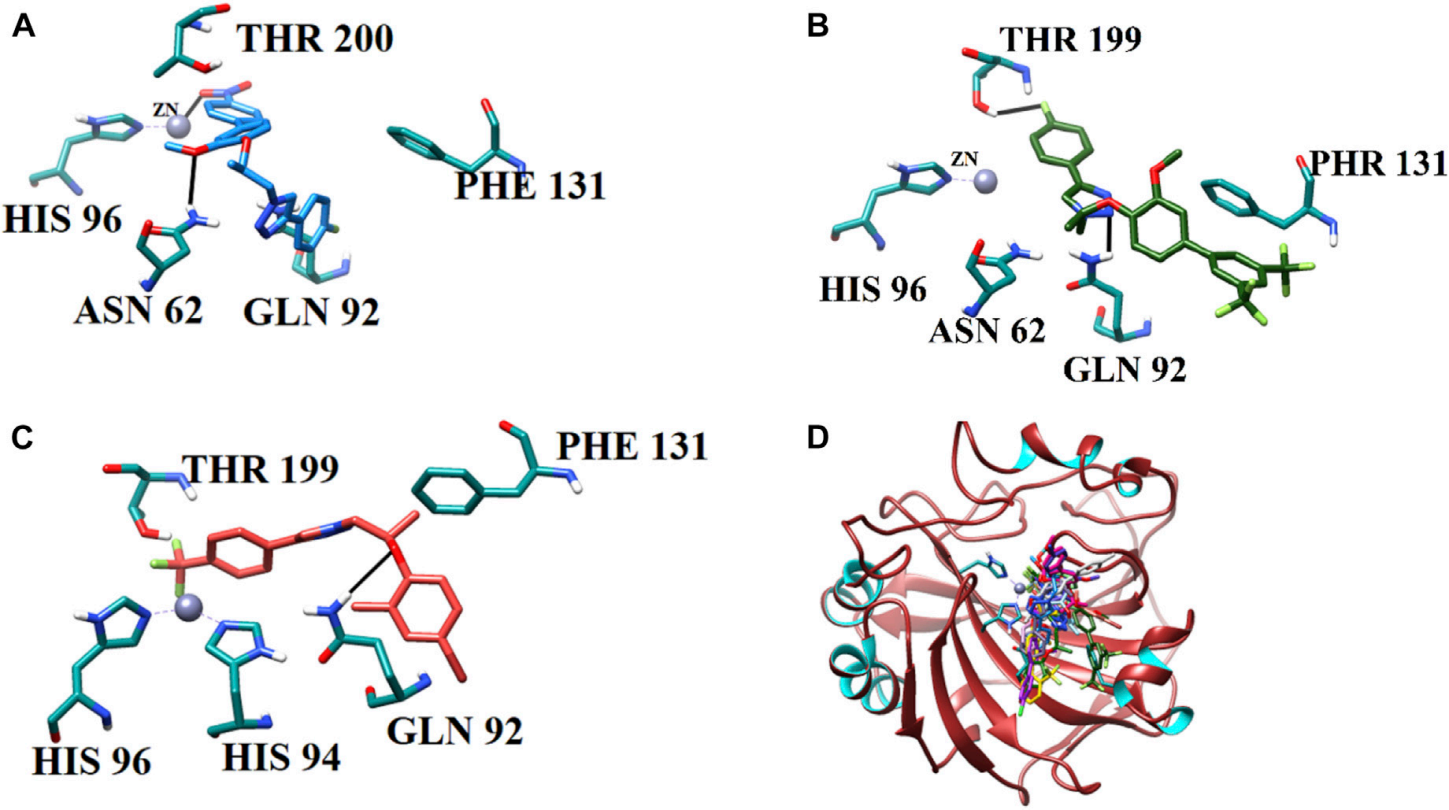
Binding orientations of **(A)** most active compound **9i** (sky blue stick model), **(B)** moderate active **9m** (green stick model), **(C)** least active **7b** (salmon stick model), and **(D)** all the active compounds (stick models) are shown in the active site of hCA-II. The active site residues are shown in cyan sticks, and H-bonds are depicted in black lines.

Compounds **7c** and **9m** exhibited moderate inhibition of hCA. The docked view of **7c** depicted that it was stabilized by the side chains of Gln92, Thr200, and Asn62 through H-bonds; moreover, the bromo group of **7c** mediated a halogen bond with the side chain of His64. Similarly, **9m** formed a halogen bond with Thr199, a H-bond with the side chain of Gln91, and a hydrophobic interaction with the phenyl ring of Phe131. Although **7a** and **7b** exhibited weak inhibition of hCA, those compounds displayed good interactions with the side chains of Thr200 and Gln92 via their triazole moieties. Additionally, **7b** also formed a halogen bond with Thr200. The binding interactions of compounds (Figure 6) and their docking scores are given in Table 5.

**TABLE 5:** Binding analysis of 1H-1,2,3-triazole analogues in the active site of hCA-II.

Compound	Binding interactions				
	Score (Kcal/mol)	Ligand atoms	Receptor atom	Bond type	Distance (Å)
**9i**	−6.41	O30	ZN	Ionic	2.08
		O12	ND2-ASN62	HBA	2.24
		N1	ND2-ASN62	HBA	2.22
**9c**	−6.82	O5	ND2-ASN62	HBA	2.15
		O12	NE2-GLN92	HBA	2.68
		F	OG1-THR199	Halogen	2.67
**9o**	−6.63	O20	NE2-HIS96	HBA	1.49
		O12	OG1-THR200	HBA	2.20
**9j**	−6.43	N17	ZN	Ionic	2.90
		O5	NE2-GLN92	HBA	1.79
		F	OG1-THR199	Halogen	2.63
**9p**	−6.13	O5	OG1-THR200	HBA	2.86
		6-ring	OG1-THR199	π–cation	1.54
**9a**	−6.73	O12	ND2-ASN62	HBA	2.05
		N1	ND2-ASN62	HBA	2.04
		O5	NE2-GLN92	HBA	1.71
		F	OG1-THR199	Halogen	2.05
**9k**	−6.09	F	NE2-HIS96	Halogen	1.55
		N17	OG1-THR199	HBA	2.66
		N16	OG1-THR200	HBA	2.07
**9f**	−6.49	O12	ND2-ASN62	HBA	2.19
		F	OG1-THR199	Halogen	2.53
		6-ring	NE2-HIS64	π–cation	1.65
**7d**	−5.15	O19	ZN	Ionic	2.33
		N1	OG-THR199	HBA	2.93
		O12	NE2-GLN92	HBA	2.53
**9n**	−6.17	O20	ZN	Ionic	1.75
		O12	OG1-THR200	HBA	1.64
		6-ring	NE2-GLN92	π–cation	2.33
**7c**	−5.59	N1	NE2-GLN92	HBA	2.13
		O5	OG1-THR200	HBA	2.52
		O12	ND2-ASN62	HBA	2.91
		Br	NE2-HIS64	Halogen	2.18
**9m**	−5.59	F	OG1-THR199	Halogen	2.36
		N17	NE2-GLN92	HBA	2.18
		6-ring	Ring-PHE131	π–π	1.58
**7a**	−4.45	N17	OG1-THR200	HBA	2.29
		O12	NE2-GLN92	HBA	2.06
**7b**	−4.24	Br	OG1-THR200	Halogen	2.14
		O12	NE2-GLN92	HBA	2.21

HBA, hydrogen bond acceptor.

### 3.5 Selectivity of 1H-1,2,3 triazole analogs against CA-II enzymes

As per selectivity of the evaluated analogues, it was found that compounds **9c**, **9k**, and **9p** are selective inhibitors of the hCA-II, whereas compounds **9d**–**9h** and **9r** are selective inhibitors of bCA-II.

## 4 Conclusion

A series of novel 1H-1,2,3-triazole analogues were synthesized (**7a**–**7d**, and **9a**–**9s**) and evaluated for their carbonic anhydrase-II inhibitory activity *in vitro*. (*S*)-(-)ethyl lactate was used as the starting material to introduce the chirality of the target molecules. The triazole moiety was prepared *via* “click” chemistry and the aryl derivatives through the Suzuki–Miyaura cross-coupling reaction. The compounds were scrutinized for their possibility to inhibit bovine and human carbonic anhydrase-II (CA-II) for the first time. All the compounds demonstrated moderate inhibitory potential against CA-II. Herein, compound **9i** was identified as a common inhibitor of both bovine and human CA-II. Furthermore, *in silico* docking analyses revealed that all the active compounds are well-accommodated in the active site of the CA-II enzyme.

## Data Availability

The original contributions presented in the study are included in the article/Supplementary Material; further inquiries can be directed to the corresponding authors.
